# An Enzyme-Free Impedimetric Sensor Based on Flower-like NiO/Carbon Microspheres for L-Glutamic Acid Assay

**DOI:** 10.3390/bios14110543

**Published:** 2024-11-09

**Authors:** Najva Sadri, Mohammad Mazloum-Ardakani, Farzaneh Asadpour, Yvonne Joseph, Parvaneh Rahimi

**Affiliations:** 1 Department of Chemistry, Faculty of Science, Yazd University, Yazd 89195-741, Iran; najva.sadri@stu.yazd.ac.ir; 2 Department of Chemistry, University of Cincinnati, 312 College Drive 404 Crosley Tower, Cincinnati, OH 45221-0172, USA; asadpofh@ucmail.uc.edu; 3 Institute of Nanoscale and Biobased Materials, Faculty of Materials Science and Technology, Technische Universität Bergakademie Freiberg, 09599 Freiberg, Germany; yvonne.joseph@esm.tu-freiberg.de

**Keywords:** non-enzymatic, electrochemical, flower-like microstructure, microsphere, L-Glutamic acid, neurotransmitter

## Abstract

This research introduces a non-enzymatic electrochemical sensor utilizing flower-like nickel oxide/carbon (fl-NiO/C) microspheres for the precise detection of L-glutamic acid (LGA), a crucial neurotransmitter in the field of healthcare and a frequently utilized food additive and flavor enhancer. The fl-NiO/C were synthesized with controllable microstructures using metal–organic frameworks (MOFs) as precursors followed by a simple calcination process. The uniformly synthesized fl-NiO/C microspheres were further characterized using Fourier transform infrared spectroscopy (FTIR), X-ray powder diffraction (XRD), energy-dispersive X-ray spectroscopy (EDX), and field emission scanning electron microscopy (FE-SEM). The fl-NiO/C was utilized as a modifier on the surface of a glassy carbon electrode, and an impedimetric sensor based on electrochemical impedance spectroscopy (EIS) was developed for the detection of LGA. The proposed sensor demonstrated excellent catalytic activity and selectivity towards LGA across a broad concentration range of 10–800 μM with a sensitivity of 486.9 µA.mM^−1^.cm^−2^ and a detection limit of 1.28 µM (S/N = 3). The sensor was also employed to identify LGA in blood plasma samples, yielding results that align with those obtained through HPLC. This achievement highlights the potential of fl-NiO/C microspheres in advancing cutting-edge biosensing applications.

## 1. Introduction

L-glutamic acid (LGA) exerts a decisive influence on a variety of brain functions as a principal excitatory neurotransmitter within the nervous system [1]. Disruption to LGA metabolism has been linked to a number of neurological disorders. These include stroke [2], Alzheimer’s disease [3], brain trauma [4], schizophrenia [5], epilepsy [6], and multiple sclerosis [7]. However, it is already established that LGA issues do not independently precipitate these conditions. To identify more efficacious treatments, it is necessary to examine the manner in which LGA interacts with other brain chemicals [8,9,10]. In addition to its neurological implications, LGA serves as a flavor enhancer in foodstuffs. Nevertheless, excessive consumption can result in adverse effects such as headaches and gastric distress [11]. Hence, monitoring LGA concentrations is imperative both in dietary and medical contexts.

Over the past two decades, various methods have been developed to quantify LGA such as chromatography [12], fluorometry [13], spectrophotometry [14], capillary electrophoresis [15], and electrochemical (bio)sensors [16,17,18]. Among them, electrochemical (bio)sensors have gained prominence in this field owing to their sensitivity, affordability, and easy operation. Electrochemical (bio)sensors designed for LGA detection can be divided into two categories: enzyme-based biosensors and non-enzymatic sensors. The first category of sensors employs the glutamate oxidase (GluOx) enzyme to produce hydrogen peroxide (H_2_O_2_) as a byproduct during the oxidative deamination of LGA in the presence of oxygen (O_2_). The production of H_2_O_2_ is directly related to the concentration of LGA [19,20]. However, enzyme-based biosensors face challenges due to the high costs of GluOx and its susceptibility to denaturation. Recently, advancements have led to the emergence of a second category of sensors that rely on the direct electrooxidation of GLA through the use of materials, which mimic the catalytic activity of GluOx. These non-enzymatic sensors are gaining popularity as an alternative to enzyme-based sensors. Transition metal/metal oxide nanomaterials and a variety of protein mediators have been employed as non-enzymatic sensing materials for the detection of LGA [21,22]. Among the materials under consideration, those based on nickel (Ni) in various forms, including nanowire [23], nickel oxide (NiO) nanoparticles [24], metal–organic frameworks (MOF), and their nanocomposites [16] have been identified as promising catalysts for LGA oxidation in an alkaline medium. This is due to their cost efficiency, electrocatalytic activity, and stability. A recent development by our research team has led to the creation of two non-enzymatic GLA sensors, utilizing two distinct synthesized materials: Ni-MOF and Ni-NiO-MOF-carbon nanocomposite. An investigation into their catalytic abilities has yielded promising results. The combination of carbon layers and MOFs forms a cavity-like network structure, which subsequently develops into a porous network. This structure enhances the electrocatalytic effect of Ni and NiO compared to the sheet-like structure of Ni-MOF [25]. This indicates that the creation of porous three-dimensional (3D) materials enhances the electrocatalytic activity of Ni-based materials.

In this context, the flower-like Ni structures have been the subject of considerable interest due to the extensive three-dimensional surface area that provides abundant space for redox reactions in electrochemical processes [26]. In particular, the fabrication of flower-like Ni derivatives based on the use of MOF as a precursor enables the creation of an adjustable porous 3D nano/micro structure [27]. Subsequently, a simple calcination process under atmospheric conditions can produce metal oxides from their corresponding MOFs, resulting in materials with inherited or identical morphologies [28].

In this investigation, we successfully synthesized porous 3D flower-like nickel oxide/carbon (fl-NiO/C) microstructures using nickel-benzene dicarboxylate (Ni-BDC) MOF as a precursor. Subsequently, nanosheets of Ni-MOF underwent a morphological transition during calcination, transforming into flower-like NiO microspheres. The fl-NiO/C were then employed to develop a straightforward non-enzymatic sensor for LGA detection on a glassy carbon electrode (GCE). The sensor was characterized using multiple electrochemical techniques. For the selective and sensitive detection of LGA across a wide linear range, electrochemical impedance spectroscopy (EIS) was used as an alternative to traditional methods like amperometry and differential pulse voltammetry. The proposed system exhibited satisfactory sensing performance, including a low detection limit, a wide dynamic range, optimal sensitivity, and suitability for measuring LGA in real samples.

## 2. Experimental Section

### 2.1. Reagent and Materials

Chemical reagents, including Benzene-1,4-dicarboxylic acid (H_2_BDC), nickel nitrate hexahydrate (Ni(NO_3_)_2_.6H_2_O), LGA, glucose (Glu), uric acid (UA), ascorbic acid (AA), tyrosine (Tyr), cysteine (Cys), glutamine (Gln), 5-hydroxytryptamine (5-HT) and sodium hydroxide (NaOH), were purchased from Sigma Aldrich. N,N-dimethylformamide (DMF) and ethanol were obtained from Merck. The chemicals were used as received without further purification. All solutions were prepared using deionized double-distilled water, and each chemical solution was freshly prepared before the experiment. Human blood plasma was obtained from the Pars Laboratory, Yazd, Iran.

### 2.2. Equipment

Fourier transform infrared spectroscopy (FT-IR) using a BRUKER EQUINOX 55 single beam spectrometer, X-ray powder Diffraction (XRD) using D8 Advance Bruker and Cu Kα radiation (λ = 1.54 Å) (Munich, Germany), energy-dispersive X-ray spectroscopy (EDS) (model EM8000F, Guangzhou, China), and field emission scanning electron microscopy (FE-SEM) using MIRA3G Tescan (Brno, Czech Republic) were employed to characterize the synthesized the fl-NiO/C. To evaluate the electrochemical properties of the prepared electrodes, we utilized an Autolab potentiostat/galvanostat (PGSTAT-302 N, Eco Chemie, and Utrecht, The Netherlands) with a standard three-electrode setup: modified GCE as the working electrode, platinum wire as the counter electrode, and Ag/AgCl/KCl (Sat.) as the reference electrode.

### 2.3. Preparation of Ni-BDC MOF as a Precursor

Ni-BDC MOF was synthesized using a typical solvothermal procedure [29]. Specifically, Ni (NO_3_)_2_.6H_2_O (0.564 g, 1.8 mmol) and H_2_BDC (0.262 g, 1.2 mmol) were separately dissolved in 10 mL of DMF at room temperature using magnetic stirring. Afterward, the Ni (NO_3_)_2_ solution was added gradually to the H_2_BDC solution and stirred for 30 min. Then, the resulting solution was placed in a Teflon-lined autoclave (50 mL) and heated at 150 °C for 12 h. The light green precipitate was collected through centrifugation, washed thrice with ethanol and water, and then, dried at 80 °C overnight.

### 2.4. Preparation of Fl-NiO/C Microsphere

The one-step calcination process was employed to synthesize fl-NiO/C microspheres. Initially, the Ni-BDC powder was ground and placed in a tubular furnace. The subsequent annealing process was carried out at 500 °C (heating rate of 5 °C/min) in the air for two hours. Following this, the black powder was collected after cooling back to room temperature.

### 2.5. Preparation of Fl-NiO/C/GCE

To prepare the modified GCE with fl-NiO/C, a GCE with a surface area of 0.03 cm^2^ was polished first using 0.3 μm alumina powder, followed by rinsing with deionized water. Thereafter, it was immersed in a solution containing water and ethanol in a ratio of 1:1, and sonicated for 10 min. Subsequently, 2 μL of the fl-NiO/C suspension (5 mg/mL in ethanol) was dropped on the clean surface of the GCE and was allowed to air dry at room temperature. The resulting sensor was designated as fl-NiO/C/GCE.

## 3. Results and Discussion

### 3.1. Material Characterization

**Figure 1:**
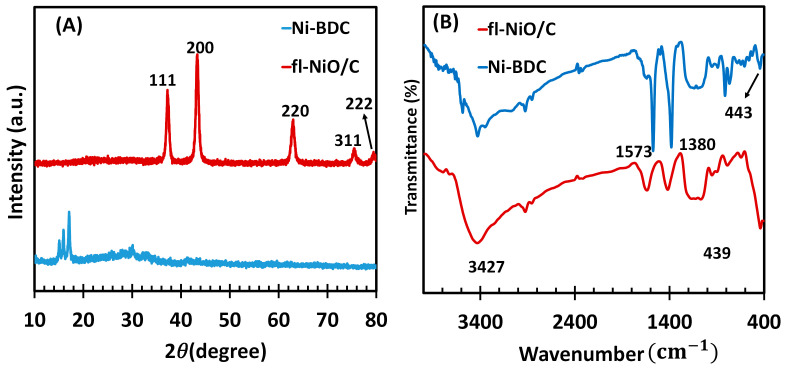
(**A**) XRD patterns and (**B**) FTIR spectrums of Ni-BDC MOF and fl-NiO/C.

To analyze the structural and compositional properties of the synthesized Ni-BDC MOF as precursor and the fl-NiO/C microsphere, FTIR and X-ray diffractometry (XRD) techniques were employed. The XRD patterns of Ni-BDC and fl-NiO/C microsphere are shown in Figure 1A. The major diffraction peaks of Ni-BDC were observed at 2θ of 15.15, 16.03, and 17.23, corresponding to the (001), (10-1), and (2-10) crystal planes, which were assigned to Ni-MOF and matched well with CCDC no. 638866, as previously reported [30]. Following the calcination of Ni-BDC, the XRD pattern of fl-NiO/C showed diffraction peaks at 37.2°, 43.3°, 63.0°, 75.6°, and 79.5°, which are attributed to the (111), (200), (220), (311), and (222) planes of face-centered cubic nickel, respectively. These peaks are consistent with the standard NiO spectrum (JCPDS, No. 47-1049) [31]. In addition, FTIR characterization of Ni-BDC and fl-NiO/C (Figure 1B) has been carried out as confirmation of their successful synthesis. The FTIR spectrum of Ni-BDC displayed two distinct peaks at approximately 1575 and 1381 cm^−1^, which correspond to the asymmetric and symmetric stretching modes of the coordinated carboxylate (–COO^−^) groups of the BDC ligands, respectively [30].

Similarly, in the case of fl-NiO/C, the absorption peaks detected at around 3427, 1600, and 1402 cm^−1^ are assigned to O−H stretching vibration and symmetric and asymmetric –COO^−^ stretching vibrations, respectively. Moreover, the peak observed at 439 cm^−1^ is attributed to Ni−O vibration absorption in fl-NiO/C, which is consistent with absorption bands of metal oxides below 1000 cm^−1^ due to interatomic vibrations [32].

**Figure 2:**
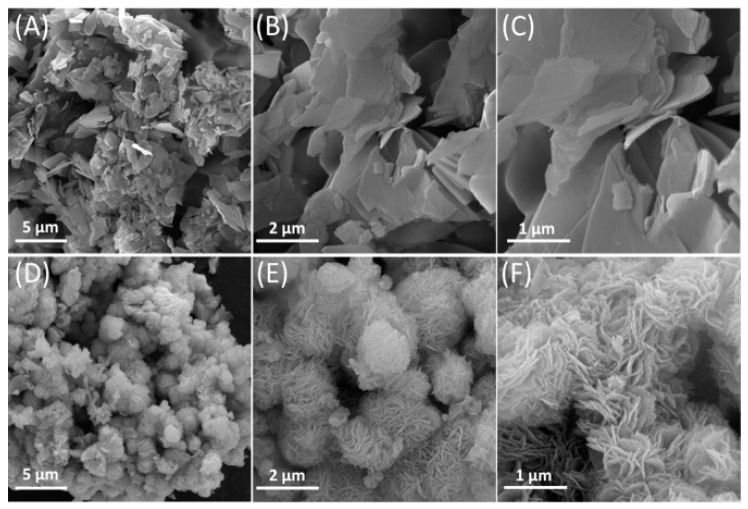
(**A**–**C**) FE-SEM images of Ni-BDC MOF and (**D**–**F**) fl-NiO/C in different magnifications.

**Figure 3:**
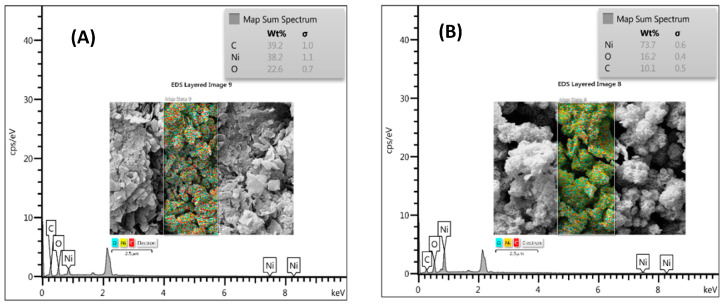
EDX mapping of (**A**) Ni-BDC MOF and (**B**) fl-NiO/C.

Further information on the morphology of the microstructures was obtained from the FE-SEM and EDS results. In the FE-SEM images, it can be observed that Ni-BDC MOF consists of numerous sheet-like structures having micro width and nano thickness, as depicted in Figure 2A–C. Upon heat treatment, these sheet-like structures entangled with each other and formed microspheres resembling flowers, as seen in Figure 2D–F. Upon magnification of the images, it was observed that the surfaces of the microspheres were significantly rough due to being covered by a large number of cross-linked nanosheets. In the following step, the EDX technique was used for the elemental analysis and chemical characterization of samples. The result of the EDX analysis showed the presence of Ni, O, and C elements in both Ni-BDC and fl-NiO/C (as shown in Figure 3A,B). Ni and C were found to have a similar distribution pattern in Ni-BDC MOF. Upon further investigation of the fl-NiO/C mapping, it was observed that the regions were rich in Ni and O and contained lower amounts of C, which could be due to the NiO coating in these areas.

### 3.2. Electrochemical Behavior of the fl-NiO/C/GCE

Cyclic voltammetry and EIS were used to study the electrochemical behavior of fl-NiO/C microspheres. To determine and compare the active surface area of fl-NiO/C/GCE with that of the bare GCE, cyclic voltammograms (CVs) of both bare and fl-NiO/C/GCE were recorded in 0.1 M KCl containing 5.0 mM [Fe (CN)_6_]^3−/4−^ as redox probe at various scan rates (Figure S1). The active surface area of the electrodes was determined by employing the Randles–Sevcik equation and utilizing the resulting slope of the plot of the current versus the square root of the scan rate (ϑ ^1/2^) [33]:

(1) $$I=2.69\times 10^{5}n^{3/2}ACD^{1/2}\vartheta^{1/2}$$

where (*I*) is the oxidation peak current, (*A*) is the surface area of the electrode, (*n*) is the electron transfer number, (*D*) is the diffusion coefficient of [Fe (CN)_6_]^3−/4−^, (*C*) is the concentration of [Fe (CN)_6_]^3−/4−^, and (ϑ) is the scan rate. The calculated surface areas for bare and modified electrodes were 0.035 and 0.093 cm^2^, respectively. This result indicates that the incorporation of fl-NiO/C significantly enhanced the electrode’s active surface area.

The surface coverage of the electrode, Γ, was estimated using the following equation, which employs the Sharp method [34], whereby the peak current can be directly proportional to the concentration of electroactive species on the electrode surface:

(2) $$I=(n^{2}F^{2}A\Gamma \vartheta )/4RT$$

The *n* represents the number of electrons involved in a reaction, while A is the active surface area (0.093 cm^2^) of the fl-NiO/C. The symbol Γ (mol/cm^2^) represents the surface coverage, and the other symbols have their usual meanings. From the slope of anodic peak currents versus scan rate (as shown in Figure S2), the surface concentration of fl-NiO/C was calculated to be Γ = 11.49 × 10^−9^ mol/cm^2^ when n = 1.

**Figure 4:**
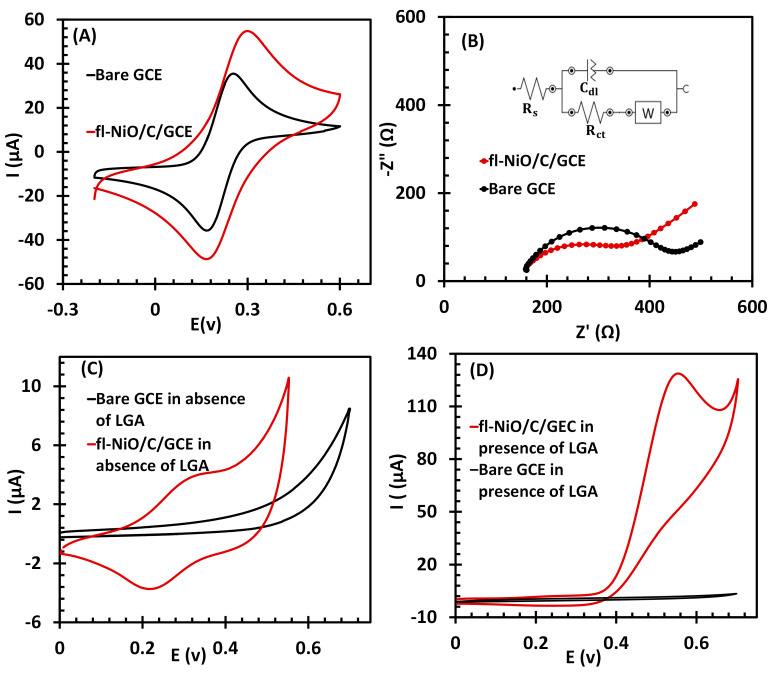
(**A**) CVs and (**B**) Nyquist plots of bare GCE and fl-NiO/C/GCE in 0.1 M KCl containing 5.0 mM [Fe(CN)_6_] ^3−/4−^, (**C**) CVs of bare GCE and fl-NiO/C/GCE in 1 M NaOH without LGA(scan rate 50 mV/s), and (**D**) CVs of Bare GCE and fl-NiO/C/GCE with 1mM LGA in 1 M NaOH at a scan rate of 50 mV/s.

To assess the changes in electrical properties and electron transfer following the modification of the GCE, and to confirm the modification’s effectiveness, CVs and EIS spectra were recorded for both the bare GCE and the fl-NiO/C/GCE in 0.1 M KCl containing 5.0 mM [Fe(CN)_6_]^3−/4−^ (Figure 4A,B, respectively). As illustrated in Figure 4A, the bare GCE exhibited a pair of characteristic redox current peaks at 0.17 V and 0.22 V, resulting from the ferro- and ferricyanide redox reactions. In comparison, the fl-NiO/C/GCE exhibited a markedly enhanced redox peak current.

EIS was conducted to confirm the CV results over a frequency range of 0.1 Hz to 100 kHz, with an applied potential of 0.22 V and a signal amplitude of 5 mV, as shown in Figure 4B. The impedance data were fitted using the Randles equivalent circuit, which appropriately models the impedance behavior of the evaluated electrochemical sensor. This circuit includes a constant phase element (CPE) to represent non-ideal capacitive behavior, often arising due to factors like surface roughness, electrode inhomogeneities, or other deviations from ideality (inset of Figure 4B). In the equivalent circuit, R_s_ represents the solution resistance, C_dl_ denotes the double layer capacitance, Z_W_ is the Warburg impedance for diffusion processes, and R_ct_ is the charge transfer resistance. The CPE is defined by parameters Y_0_ (or Q) (admittance at 1 rad/s) and n (which indicates the extent of deviation from ideal capacitance). For our system, the value of Y_0_ is 4.39 μM ho⋅s^n^, where n is 0.767.

The Nyquist plots (Figure 4B) reveal distinct semicircles in the high-frequency region for each electrode, indicative of R_ct_. The linear portion in the low-frequency region is attributed to diffusion-limited processes. Notably, the R_ct_ value for the bare glassy carbon electrode (GCE) is 326.1 Ω, which decreases to 218.3 Ω when modified with fl-NiO/C microspheres. This reduction in R_ct_ reflects the role of the porous 3D structure of fl-NiO/C microspheres in increasing the electrochemically accessible surface area. The modified structure enables additional electron transfer pathways, thus improving electrical conductivity and facilitating faster electron exchange.

The electrocatalytic behavior of fl-NiO/C/GCE towards LGA was analyzed in 1 M NaOH solution via cyclic voltammetry, employing a scan rate of 50 mV/s. As shown in Figure 4C, in the absence of LGA, the bare GCE is relatively inactive within a specific potential range and does not show any significant signals. On the other hand, the fl-NiO/C/GCE exhibits clear redox peaks at potentials of 0.32 V (anodic) and 0.23 V (cathodic). Two distinct theories have been proposed to explain the observed redox peaks. One theory suggests that these peaks arise from the oxidation of Ni^2+^ to Ni^3+^ and its corresponding reduction [35]. An alternative interpretation, as proposed by Jamal et al., indicates that the anodic peak results from the oxidation of NiO to NiOOH, while the cathodic peak represents its subsequent reduction [24]. The elemental characterization of fl-NiO/C, obtained from EDX and XRD analyses, showed that the microspheres are predominantly composed of NiO, supporting the second hypothesis that the electrochemical behavior is due to the conversion of NiO to NiOOH and vice versa. Figure 4D shows the performance of bare GCE and fl-NiO/C/GCE in the presence of 1 mM LGA. Upon the addition of LGA, the bare GCE did not exhibit any response to LGA, as LGA cannot readily undergo the electron transfer at the surface of a bare electrode, even when subjected to a significantly higher voltage. In contrast, the presence of LGA resulted in a significant increase in the anodic peak current (I_pa_) of fl-NiO/C/GCE, from 4 to approximately 130 µA. Conversely, the cathodic peak becomes undetectable. This suggests that fl-NiO/C is involved in a catalytic process in alkaline media, as previously observed for nickel and nickel oxide-modified electrodes [36,37,38]. As observed, the anodic peak has undergone a slight shift to a higher potential, which is attributed to the restricted diffusion of LGA towards the electrode, a phenomenon that has been previously reported [24,39,40].

The mechanism of LGA oxidation by fl-NiO/C microspheres can be illustrated through the following reactions [17,23,24]:

(3) $$f\mathrm{l-}NiO/C+H_{2}O+{2OH}^{-}↔f\mathrm{l-}Ni{\left(OH\right)}_{2}/C+{2OH}^{-}$$

(4) $${f\mathrm{l-}Ni(OH)}_{2}/C+{OH}^{-}↔f\mathrm{l-}NiOOH/C+H_{2}O+e$$

(5) $$f\mathrm{l-}NiOOH/C+\mathrm{L-}glutamic\;acid\to Oxogluartate+f\mathrm{l-}Ni{\left(OH\right)}_{2}/C$$

**Figure 5:**
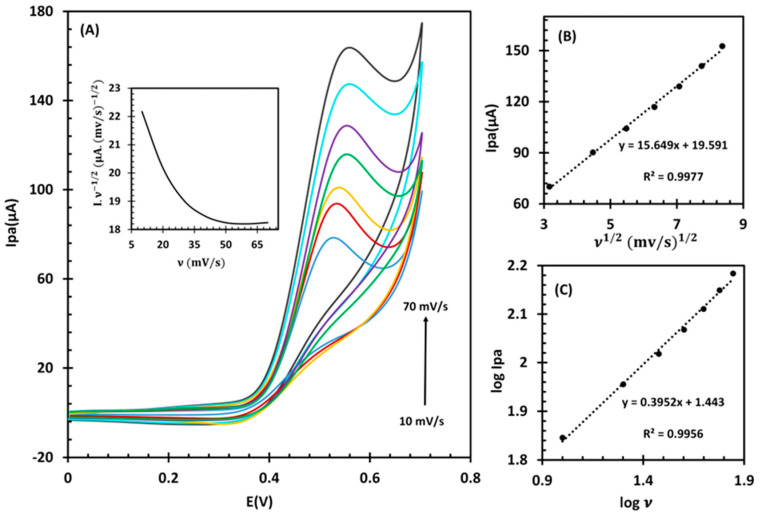
(**A**) CVs of fl-NiO/C/GCE in 1 M NaOH containing 1mM LGA at different scan rates (10, 20, 30, 40, 50, 60, and 70 mV/s). Inset: variation of normalized current (I/υ^1/2^) vs. scan rate. (**B**) The linear relationships of I versus υ ^1/2^ and (**C**) log I_p_ versus log υ.

To obtain more information about the electrochemical mechanism, a series of CVs were conducted at different scan rates, ranging from 10 to 70 mV/s (Figure 5A). As the scan rates increased, the oxidation peak potential was observed to shift slightly towards a more positive potential. This shift confirmed that the electrochemical reaction was kinetically limited. In correspondence with the Randles–Sevick equation [41], a plot of I_pa_ versus the square root of scan rate (υ ^1/2^), in the range of 10–70 mV/s, was obtained (Figure 5B). The linear enhancement of the LGA oxidation current to the υ ^1/2^ and the linear relationship between log I and log υ with a slope value of 0.39 (that is, near the theoretical value of 0.50), as displayed in Figure 5C, imply that the reaction was controlled by diffusion. Additionally, a graph of the normalized current (I/υ ^1/2^) against the scan rate (inset shown in Figure 5A) displayed the typical EC’ process characteristics [33]. In order to gain insights into the kinetics of the electrooxidation process, particularly the electron transfer coefficient (α) and the number of electrons (n) involved in the LGA oxidation reaction, the Tafel plot of E vs log I_pa_ was generated. This plot was derived from the rising portion of the CV at a scan rate of 10 mV/s. At this scan rate, it is assumed that the electro-oxidation process is influenced solely by electron transfer kinetics without the effects of mass transport limitations. By employing the Tafel slope equation (slope = 2.3RT/(nf(1 − α))), the combined value of n(1 − α) was determined to be 0.57. Given the documented range for α between 0.3 and 0.7 [42,43], it is reasonable to estimate n = 1 and α = 0.43. This transfer coefficient aligns well with the typical range for electrochemical reactions, thereby supporting a one-electron transfer mechanism in the LGA electrooxidation process [44].

### 3.3. Chronoamperometric Measurements

**Figure 6:**
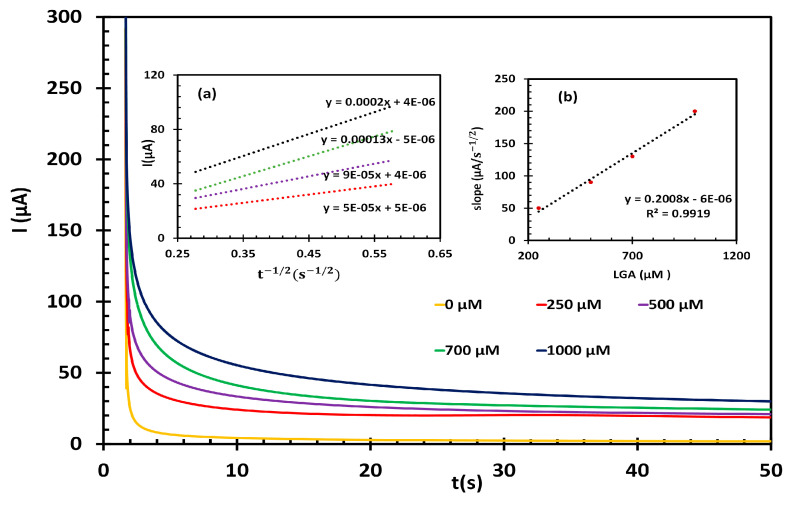
(A) Chronoamperograms of fl-NiO/C/GCE in 1 M NaOH solution containing different concentrations of LGA 0, 250, 500, 300, 700, and 1000 μM. Insets: (**a**) plots of I vs. t^−1/2^ obtained from chronoamperograms and (**b**) plot of the slope of the straight lines from inset (**a**) against the LGA concentration).

Chronoamperometry experiments were further conducted to estimate the catalytic rate constant (k) and the diffusion coefficient (D) of LGA at the fl-NiO/C/GCE surface. The current time profiles were obtained for varying concentrations of LGA (0, 250, 500, 700, and 1000 μM) in 1M NaOH solution (Figure 6). The working electrode’s potential was adjusted to 0.6 V. According to the Cottrell equation, the D of LGA can be determined by plotting the current (I) versus the square root of time (t^1/2^). The experimental plots for different concentrations of LGA are shown in Figure 6 inset (a), which have been fitted accordingly. After plotting the slopes of the lines against the LGA concentration (Figure 6 inset b), the resulting slope enabled the calculation of an average value for D, which was found to be 1.49 × 10^−6^ cm^2^.s^−1^. Moreover, based on the Galus method, k for the oxidation of LGA at the fl-NiO/C/GCE surface was calculated using the following equation:

(6) $$I_{C}/I_{L}=\gamma^{\left(1/2\right)}[(\pi^{\left(1/2\right)}erf(\gamma^{\left(1/2\right)})+exp(-\gamma ))/\gamma^{\left(1/2\right)}]$$

where $I_{C}$ is the catalytic current of LGA at the fl-NiO/C/GCE, $I_{L}$ represents the limited current in the absence of LGA, and $\gamma =k{.C}_{b}.t$ where $C_{b}\;$is the bulk concentration of LGA. When γ approaches 2, the error function is nearly equal to 1. In such cases, the aforementioned equation can be simplified as follows:

(7) $$I_{C}/I_{L}=\pi^{\left(1/2\right)}\gamma^{\left(1/2\right)}=\pi^{\left(1/2\right)}{\left(kC_{b}t\right)}^{\left(1/2\right)}$$

To determine the rate constant value of the catalytic process, $I_{C}/I_{L}$ was plotted against t ^1/2^ for various concentrations of LGA, as shown in Figure S4. The slopes of the resultant plots were then used to calculate an average value for k, which was found to be 5.212 × 10^3^ M^−1^.s^−1^. This value confirms that fl-NiO/C film provides rapid electron transfer for the oxidation of LGA.

### 3.4. Electrochemical Impedance Spectroscopy (EIS) Measurements

**Figure 7:**
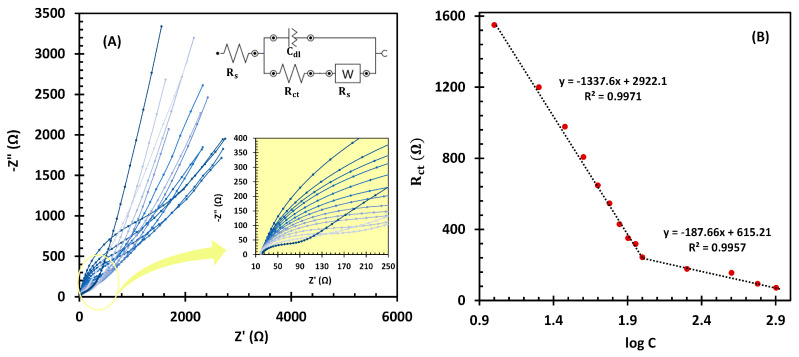
(**A**) EIS curves of different concentrations of LGA (10.0, 20.0, 30.0, 40.0, 50.0, 60.0, 70.0, 80.0, 90.0, 100.0, 200.0, 400.0, 600.0, and 800.0 µM. Inset: high magnifications of EIS curves). (**B**) The linear relationship between the logarithm of the LGA concentrations and R_ct_.

EIS has recently emerged as a promising alternative to amperometry and differential pulse voltammetry for the development of sensitive, enzyme-free impedimetric biosensors, particularly for biomolecule detection at low concentrations [45,46]. The sensor functions by tracking changes in R_ct_ in relation to the target molecule concentration, offering a reliable approach to the detection of analytes. In this study, EIS was further utilized to evaluate the effectiveness of fl-NiO/C/GCE as an LGA impedimetric sensor in a frequency range of 100 kHz to 0.1 Hz. The oscillation amplitude was set to 10 mV, and the working potential was adjusted at 0.45 V vs Ag/AgCl. The Nyquist plots of the fl-NiO/C/GCE in 1 M NaOH for various LGA concentrations are shown in Figure 7A. As can be seen, the R_ct_ magnitude decreases gradually with the addition of concentrations ranging from 10.0 to 800.0 µM LGA. The inset of Figure 7A illustrates the modified Randles equivalent circuit (MREC), which is used for EIS data fitting. The calibration curve plotting the logarithmic effect of LGA concentration addition on the R_ct_ response of the fl-NiO/C/GCE sensor is depicted in Figure 7B.

Accordingly, the linear fit for the R_ct_ of LGA follows the equation R_ct_ = −1337.6 (log [LGA]) + 2922.1 in the 10–100 µM and R_ct_ = −187.66 (log [LGA]) + 615.21 in the 100–800 µM. The detection limit and sensitivity in the lower concentration ranges were obtained as 1.28 µM (S/N = 3) and 428.98 µA.mM^−1^.cm^−2^, respectively. These results demonstrate that EIS is not only comparable to commonly used methods such as amperometry and differential pulse voltammetry, but also offers greater sensitivity, with a lower detection limit and a broader linear concentration range towards LGA detection. A comparison of the data presented in Table 1 [19,21,23,24,47,48] shows that the fl-NiO/C/GCE impedimetric sensor exhibits superior analytical performance compared to previous studies.

### 3.5. Interference Study of Biosensor

**Figure 8:**
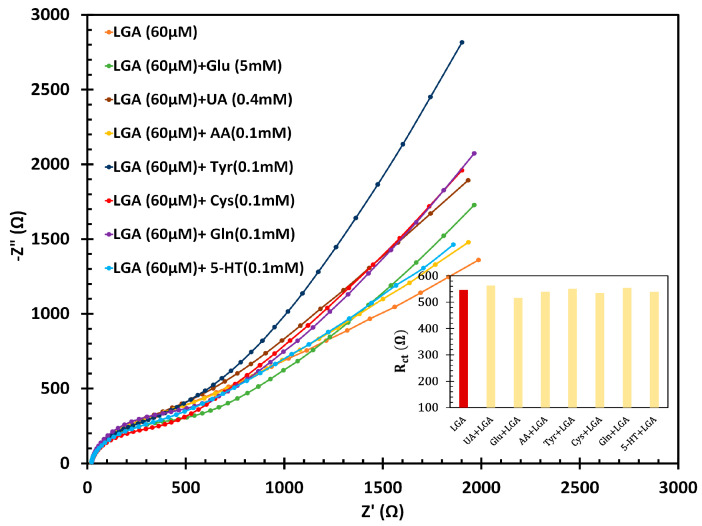
Interference study of fl-NiO/C/GCE in 1 M NaOH in presence of 5 mM of Glu, 0.1 mM of AA, and 0.4 mM of UA alongside 60 µM of LGA.

The sensor’s selectivity was evaluated by testing the electrode in the presence of common oxidative species such as Glu, AA, and UA, which are often found alongside LGA in biological environments. Additionally, structurally related biomolecules, including Tyr, Cys, Gln, and neurotransmitters like 5-HT, were also tested. The impedimetric responses of the fl-NiO/C/GCE electrode in a 1M NaOH solution included standard concentrations of these interfering species alongside LGA (60 µM). Using the equation (R_ct_(mix)−R_ct_(LGA))/R_ct_(LGA), we assessed the impact of these potential interferences on LGA recognition. The highest relative standard deviation (RSD%) for interference was between 1.3% and 5.7%, demonstrating the sensor’s accuracy in detecting LGA (as shown in Figure 8).

### 3.6. Stability, Reproducibility, and Repeatability

The stability of the fl-NiO/C/GCE in detecting 200 μM LGA was examined using amperometry through monitoring the sensor’s response for 600 s before and after the introduction of LGA. The findings illustrated in Figure S5A indicate that the sensor maintained nearly 97% of its initial current response to LGA even after 1000 s, signifying satisfactory stability. Additionally, to confirm the stability and effectiveness of the electrode modification, up to 100 CVs of fl-NiO/C/GCE were recorded (Figure S5B). As shown, approximately 65% of the redox peak current of fl-NiO/C/GCE was retained even after 100 CV cycles, indicating that the fl-NiO/C modification remained adhered to the electrode surface. Subsequent evaluation after 10 days (sensor remained at 4 °C) demonstrated a consistent response with low relative standard deviation (RSD)(Figure S6). Furthermore, five distinct electrodes were fabricated, and their sensing responses towards LGA were assessed using CV to evaluate reproducibility (Figure S7). The obtained RSD of 2.5% highlights the sensor’s good reproducibility. Additionally, the prepared fl-NiO/C/GCE sensor was immersed in a 1M NaOH solution with 0.8 mM LGA, and the R_ct_ was evaluated over 10 consecutive measurements. The resulting standard deviation from these measurements was 1.7%, indicating excellent repeatability.

### 3.7. Utilizing the Sensor for Real Sample Analysis

To validate the practical feasibility of the fl-NiO/C/GCE sensor, it was employed to detect LGA in blood plasma samples using EIS method. Three blood plasma samples (I–III) with known LGA concentrations, determined via high-performance liquid chromatography (HPLC), were obtained from a local clinical laboratory in Yazd, Iran. All serum samples were, indeed, treated with NaOH solution prior to measurement. The LGA levels were established through a calibration curve, with the recovery rates for samples spanning from 98 to 102%, as indicated in Table 2.

Upon statistic studies and juxtaposing the experimental t values with the critical t value (t_crit_ = 2.89 at confidence level of 99%), it became apparent that there existed no significant disparity between the results obtained from the proposed impedimetric sensor and those obtained from the HPLC method, demonstrating the sensor’s utility for analyzing real samples. Impedimetric diagrams of blood plasma samples are shown in Figure S8.

## 4. Conclusions

The study successfully developed 3D porous flower-like microspheres of NiO/C through a direct calcination method of Ni-BDC MOF, which were subsequently utilized as a non-enzymatic sensing material to modify the surface of GCE for the detection of LGA. The electrochemical results indicated that the uniformly structured 3D fl-NiO/C microspheres provided a larger electrochemically accessible surface area for LGA, effectively enhancing the electron transfer kinetics. Furthermore, the proposed impedimetric sensor demonstrated remarkable potential for practical LGA detection, showcasing exceptional sensitivity, low detection limits, and a wide linear range. Additionally, the developed non-enzymatic sensor exhibited notable reproducibility, selectivity, and stability, along with successful application in real samples, confirming its potential in biosensing.

## Figures and Tables

**Table 1:** Comparison of different non-enzymatic sensors for L-glutamic acid detection.

Electrodes	Detection Method	Linearity Range (mM)	Sensitivity (μA mM^−1^ cm^−2^)	LOD ^2^ (μM)	Enzyme	References
**NiNAE ^1^**	I–V	0.50–8.00	65.0	135.00	no enzyme	[23]
**NiO/GCE**	Amperometric	1.00–8.00	11.00	272.00	no enzyme	[24]
**Au@MoS_2_/Chitosan/** **GCE**	DPV ^3^	0.00005–0.20	---	0.03	no enzyme	[48]
**PPy ^4^/Nafion/Chitosan/** **GlutOx ^5^**	Amperometric	0.01−0.88	38	2.5	GlutOx	[47]
**sGlutOx/cMWCNT ^6^/AuNP/Chitosan**	Cyclic voltammetry	0.005–0.5	155	1.6	GlutOx	[19]
**GlutOx/APTES ^7^/** **ta-C/P ^8^**	Amperometric	0.01–0.5	2.9	10.0	GlutOx	[21]
**fl-NiO/C/GCE**	EIS	0.01–0.80	486.98	1.28	no enzyme	This work

^1^ Nickel nanowire array, ^2^ limit of detection, ^3^ differential pulse voltammetry, ^4^ polypyrrole, ^5^ glutamate oxidase, ^6^ multi-walled carbon nanotubes, ^7^ (3-aminopropyl)triethoxysilane, and ^8^ tetra-hedral amorphous carbon.

**Table 2:** Determination of LGA level in blood plasma sample using the fl-NiO/C/GCE electrode (in 1 M NaOH solution).

Blood Plasma Sample	Method	Found by This Sensor (μM)	Found by HPLC (μM)	Recovery%	t_experimental_
**I**	EIS	89.1	90.1	98.91	1.21
**II**	EIS	46.0	45.1	102.13	1.27
**III**	EIS	30.9	31.3	99.55	1.83

## Data Availability

The data supporting this article have been included as part of the Supplementary Information.

## Supplementary Materials

The following supporting information can be downloaded at: https://www.mdpi.com/article/10.3390/bios14110543/s1, Figure S1. CVs of fl-NiO/C/GCE in 5.0 mM [Fe (CN)_6_]^3−/4−^ in 0.1 M KCl at a scan rate of 20–800 mV/s (A) and the plot of I versus υ^1/2^ (B). CVs of bare GCE in 5.0 mM [Fe (CN)_6_]^3−/4−^ in 0.1 M KCl at a scan rate of 20–800 mV/s (C) and the plot of I versus υ^1/2^ (D). Figure S2. Cyclic voltammogram of fl-NiO/C/GCE in 1 M NaOH solution at various scan rate 10–100 mV/s. (Inset: Plot of I versus υ). Figure S3. CVs of fl-NiO/C/GCE in 1 mM LGA in 1 M NaOH at a scan rate of 10 mV/s (A). Tafel plot E vs log I (B). Figure S4. The IC/IL vs. t^1/2^ plot at different concentrations of LGA (250, 500, 700, and 1000 μM) (A). The plot of the slope of the (IC/IL vs.t^1/2^) vs. C^1/2^ (B). Figure S5. Amprometric response of fl-NiO/C/GCE 600 s before and after injection of 200 µL GLA (A) and CVs of fl-NiO/C/GCE in 1 M NaOH without LGA for 100 cycles, scan rate 50 mv/s (B). Figure S6. Stability of the fl-NiO/C/GCE during different times. Figure S7. CVs of 5 prepared fl-NiO/C/GCE in 1mM LGA in 1 M NaOH at a scan rate of 50 mV/s. Figure S8. EIS responses of the fl-NiO/C/GCE towards sample (I–III).

## References

[1] Schultz J., Uddin Z., Singh G., Howlader M.M. (2020). Glutamate Sensing in Biofluids: Recent Advances and Research Challenges of Electrochemical Sensors. Analyst.

[2] Campos F., Sobrino T., Ramos-Cabrer P., Argibay B., Agulla J., Pérez-Mato M., Rodríguez-González R., Brea D., Castillo J. (2011). Neuroprotection by Glutamate Oxaloacetate Transaminase in Ischemic Stroke: An Experimental Study. J. Cereb. Blood Flow Metab..

[3] Walton H.S., Dodd P.R. (2007). Glutamate–Glutamine Cycling in Alzheimer’s Disease. Neurochem. Int..

[4] Yi J.H., Hazell A.S. (2006). Excitotoxic Mechanisms and the Role of Astrocytic Glutamate Transporters in Traumatic Brain Injury. Neurochem. Int..

[5] Volk D.W., Austin M.C., Pierri J.N., Sampson A.R., Lewis D.A. (2000). Decreased Glutamic Acid Decarboxylase67 Messenger RNA Expression in a Subset of Prefrontal Cortical γ-Aminobutyric Acid Neurons in Subjects with Schizophrenia. Arch. Gen. Psychiatry.

[6] Liimatainen S., Peltola M., Sabater L., Fallah M., Kharazmi E., Haapala A.M., Dastidar P., Knip M., Saiz A., Peltola J. (2010). Clinical Significance of Glutamic Acid Decarboxylase Antibodies in Patients with Epilepsy. Epilepsia.

[7] Stojanovic I.R., Kostic M., Ljubisavljevic S. (2014). The Role of Glutamate and Its Receptors in Multiple Sclerosis. J. Neural Transm..

[8] De Bartolomeis A., Buonaguro E.F., Iasevoli F. (2012). Serotonin–Glutamate and Serotonin–Dopamine Reciprocal Interactions as Putative Molecular Targets for Novel Antipsychotic Treatments: From Receptor Heterodimers to Postsynaptic Scaffolding and Effector Proteins. Psychopharmacology.

[9] Asadpour F., Mazloum-Ardakani M. (2022). Electro-Assisted Self-Assembly of Mesoporous Silica Thin Films: Application to Electrochemical Sensing of Glutathione in the Presence of Copper. J. Solid State Electrochem..

[10] Asadpour F., Zhang X.W., Mazloum-Ardakani M., Mirzaei M., Majdi S., Ewing A.G. (2021). Vesicular Release Dynamics Are Altered by the Interaction between the Chemical Cargo and Vesicle Membrane Lipids. Chem. Sci..

[11] Jinap S., Hajeb P. (2010). Glutamate. Its Applications in Food and Contribution to Health. Appetite.

[12] Monge-Acuña A.A., Fornaguera-Trías J. (2009). A High Performance Liquid Chromatography Method with Electrochemical Detection of Gamma-Aminobutyric Acid, Glutamate and Glutamine in Rat Brain Homogenates. J. Neurosci. Methods.

[13] Chapman J., Zhou M. (1999). Microplate-Based Fluorometric Methods for the Enzymatic Determination of l-Glutamate: Application in Measuring l-Glutamate in Food Samples. Anal. Chim. Acta.

[14] Buck K., Voehringer P., Ferger B. (2009). Rapid Analysis of GABA and Glutamate in Microdialysis Samples Using High Performance Liquid Chromatography and Tandem Mass Spectrometry. J. Neurosci. Methods.

[15] Lada M.W., Vickroy T.W., Kennedy R.T. (1997). High Temporal Resolution Monitoring of Glutamate and Aspartate in Vivo Using Microdialysis On-Line with Capillary Electrophoresis with Laser-Induced Fluorescence Detection. Anal. Chem..

[16] Hussain M.M., Rahman M.M., Asiri A.M., Awual M.R. (2016). Non-Enzymatic Simultaneous Detection of l-Glutamic Acid and Uric Acid Using Mesoporous Co_3_O_4_ Nanosheets. RSC Adv..

[17] Shadlaghani A., Farzaneh M., Kinser D., Reid R.C. (2019). Direct Electrochemical Detection of Glutamate, Acetylcholine, Choline, and Adenosine Using Non-Enzymatic Electrodes. Sensors.

[18] Scoggin J.L., Tan C., Nguyen N.H., Kansakar U., Madadi M., Siddiqui S., Arumugam P.U., DeCoster M.A., Murray T.A. (2019). An Enzyme-Based Electrochemical Biosensor Probe with Sensitivity to Detect Astrocytic versus Glioma Uptake of Glutamate in Real Time in Vitro. Biosens. Bioelectron..

[19] Batra B., Pundir C.S. (2013). An Amperometric Glutamate Biosensor Based on Immobilization of Glutamate Oxidase onto Carboxylated Multiwalled Carbon Nanotubes/Gold Nanoparticles/Chitosan Composite Film Modified Au Electrode. Biosens. Bioelectron..

[20] Soldatkina O.V., Soldatkin O.O., Kasap B.O., Kucherenko D.Y., Kucherenko I.S., Kurc B.A., Dzyadevych S.V. (2017). A Novel Amperometric Glutamate Biosensor Based on Glutamate Oxidase Adsorbed on Silicalite. Nanoscale Res. Lett..

[21] Kaivosoja E., Tujunen N., Jokinen V., Protopopova V., Heinilehto S., Koskinen J., Laurila T. (2015). Glutamate Detection by Amino Functionalized Tetrahedral Amorphous Carbon Surfaces. Talanta.

[22] Özel R.E., Ispas C., Ganesana M., Leiter J.C., Andreescu S. (2014). Glutamate Oxidase Biosensor Based on Mixed Ceria and Titania Nanoparticles for the Detection of Glutamate in Hypoxic Environments. Biosens. Bioelectron..

[23] Jamal M., Hasan M., Mathewson A., Razeeb K.M. (2013). Disposable Sensor Based on Enzyme-Free Ni Nanowire Array Electrode to Detect Glutamate. Biosens. Bioelectron..

[24] Jamal M., Chakrabarty S., Shao H., McNulty D., Yousuf M.A., Furukawa H., Khosla A., Razeeb K.M. (2018). A Non Enzymatic Glutamate Sensor Based on Nickel Oxide Nanoparticle. Microsyst. Technol..

[25] Alizadeh Z., Mazloum-Ardakani M., Asadpour F., Yavari M. (2023). Highly Efficient Enzyme-Free Glutamate Sensors Using Porous Network Metal–Organic Framework-Ni-NiO-Ni-Carbon Nanocomposites. ACS Appl. Mater. Interfaces.

[26] Li Q., Chen Y., Yang T., Lei D., Zhang G., Mei L., Chen L., Li Q., Wang T. (2013). Preparation of 3D Flower-like NiO Hierarchical Architectures and Their Electrochemical Properties in Lithium-Ion Batteries. Electrochim. Acta.

[27] Qiu Y., Yang H., Cheng Y., Lin Y. (2022). MOFs Derived Flower-like Nickel and Carbon Composites with Controllable Structure toward Efficient Microwave Absorption. Compos. Part A Appl. Sci. Manuf..

[28] Guo W., Sun W., Lv L.P., Kong S., Wang Y. (2017). Microwave-Assisted Morphology Evolution of Fe-Based Metal-Organic Frameworks and Their Derived Fe_2_O_3_ Nanostructures for Li-Ion Storage. ACS Nano.

[29] Gumilar G., Kaneti Y.V., Henzie J., Chatterjee S., Na J., Yuliarto B., Nugraha N., Patah A., Bhaumik A., Yamauchi Y. (2020). General Synthesis of Hierarchical Sheet/Plate-like M-BDC (M = Cu, Mn, Ni, and Zr) Metal–Organic Frameworks for Electrochemical Non-Enzymatic Glucose Sensing. Chem. Sci..

[30] Yang J., Zheng C., Xiong P., Li Y., Wei M. (2014). Zn-Doped Ni-MOF Material with a High Supercapacitive Performance. J. Mater. Chem. A.

[31] Goel R., Jha R., Ravikant C. (2020). Investigating the Structural, Electrochemical, and Optical Properties of p-Type Spherical Nickel Oxide (NiO) Nanoparticles. J. Phys. Chem. Solids.

[32] Sudha V., Senthil Kumar S.M., Thangamuthu R. (2018). Synthesis and Characterization of NiO Nanoplatelet and Its Application in Electrochemical Sensing of Sulphite. J. Alloys Compd..

[33] Bard A.J., Faulkner L.R., Swain E., Robey C. (1995). Fundamentals and Applications.

[34] Sharp M., Petersson M., Edström K. (1980). A Comparison of the Charge Transfer Kinetics between Platinum and Ferrocene in Solution and in the Surface Attached State. J. Electroanal. Chem. Interfacial Electrochem..

[35] Trafela Š., Zavašnik J., Šturm S., Rožman K.Ž. (2019). Formation of a Ni(OH)_2_/NiOOH Active Redox Couple on Nickel Nanowires for Formaldehyde Detection in Alkaline Media. Electrochim. Acta.

[36] Wang J., Zhao Q., Hou H., Wu Y., Yu W., Ji X., Shao L. (2017). Nickel Nanoparticles Supported on Nitrogen-Doped Honeycomb-like Carbon Frameworks for Effective Methanol Oxidation. RSC Adv..

[37] Youcef M., Hamza B., Nora H., Walid B., Salima M., Ahmed B., Malika F., Marc S., Christian B., Wassila D. (2022). A Novel Green Synthesized NiO Nanoparticles Modified Glassy Carbon Electrode for Non-Enzymatic Glucose Sensing. Microchem. J..

[38] Mazloum-Ardakani M., Amin-Sadrabadi E., Khoshroo A. (2016). Enhanced Activity for Non-Enzymatic Glucose Oxidation on Nickel Nanostructure Supported on PEDOT:PSS. J. Electroanal. Chem..

[39] Amjad M., Pletcher D., Smith C. (1977). The Oxidation of Alcohols at a Nickel Anode in Alkaline T-Butanol/Water Mixtures. J. Electrochem. Soc..

[40] Raveendran A., Chandran M., Dhanusuraman R. (2023). A Comprehensive Review on the Electrochemical Parameters and Recent Material Development of Electrochemical Water Splitting Electrocatalysts. RSC Adv..

[41] Cordeiro C.A., De Vries M.G., Cremers T.I.F.H., Westerink B.H.C. (2016). The Role of Surface Availability in Membrane-Induced Selectivity for Amperometric Enzyme-Based Biosensors. Sens. Actuators B Chem..

[42] Rahimi P., Rafiee-Pour H.A., Ghourchian H., Norouzi P., Ganjali M.R. (2010). Ionic-Liquid/NH2-MWCNTs as a Highly Sensitive Nano-Composite for Catalase Direct Electrochemistry. Biosens. Bioelectron..

[43] Lee H.Y., Cho W.S., Oh S.M. (1998). Active Reaction Sites and Oxygen Reduction Kinetics on La1-xSrxMnO_3_ + δ (x = 0.1–0.4)/YSZ (Yttria-Stabilized Zirconia) Electrodes for Solid Oxide Fuel Cells. Bull. Korean Chem. Soc..

[44] Analytical Sciences Digital Library Reversibility: Chemical vs. Electrochemical. *LibreTexts*. https://chem.libretexts.org/@go/page/61288?pdf.

[45] Rinaldi A.L., Carballo R. (2016). Impedimetric Non-Enzymatic Glucose Sensor Based on Nickel Hydroxide Thin Film onto Gold Electrode. Sens. Actuators B Chem..

[46] Zarei A., Hatefi-Mehrjardi A., Karimi M.A., Mohadesi A. (2022). Impedimetric Glucose Biosensing Based on Drop-Cast of Porous Graphene, Nafion, Ferrocene, and Glucose Oxidase Biocomposite Optimized by Central Composite Design. J. Electroanal. Chem..

[47] Tseng T.T.C., Yao J., Chan W.C. (2013). Selective Enzyme Immobilization on Arrayed Microelectrodes for the Application of Sensing Neurotransmitters. Biochem. Eng. J..

[48] Devi R., Gogoi S., Barua S., Sankar Dutta H., Bordoloi M., Khan R. (2019). Electrochemical Detection of Monosodium Glutamate in Foodstuffs Based on Au@MoS_2_/Chitosan Modified Glassy Carbon Electrode. Food Chem..

