# Supplementary material for: An Enzyme-Free Impedimetric Sensor Based on Flower-like NiO/Carbon Microspheres for L-Glutamic Acid Assay
Source: Biosensors (Basel). 2024 Nov 9;14(11):543. doi: 10.3390/bios14110543 (PMC11591706; doi:10.3390/bios14110543)
Supplement: Supplementary file 1 [file biosensors-14-00543-s001.zip › biosensors-3287215-supplementary.pdf]

Supporting information

An Enzyme-Free Impedimetric Sensor Based on Flower-Like NiO/Carbon Microspheres for L-Glutamic Acid Assay

Najva Sadri [1], Mohammad Mazloum-Ardakani [1,*], Farzaneh Asadpour [2], Yvonne Joseph [3] and Parvaneh Rahimi [3,*]

[1] Department of Chemistry, Faculty of Science, Yazd University, Yazd 89195-741, Iran; najva.sadri@stu.yazd.ac.ir

[2] Department of Chemistry, University of Cincinnati, 312 College Drive 404 Crosley Tower, Cincinnati, OH 45221-0172, USA; asadpofh@ucmail.uc.edu

[3] Institute of Nanoscale and Biobased Materials, Faculty of Materials Science and Technology, Technische Universität Bergakademie Freiberg, 09599 Freiberg, Germany; yvonne.joseph@esm.tu-freiberg.de

* Correspondence: mazloum@yazd.ac.ir (M.M.-A.); Parvaneh.Rahimi@esm.tu-freiberg.de (P.R.)

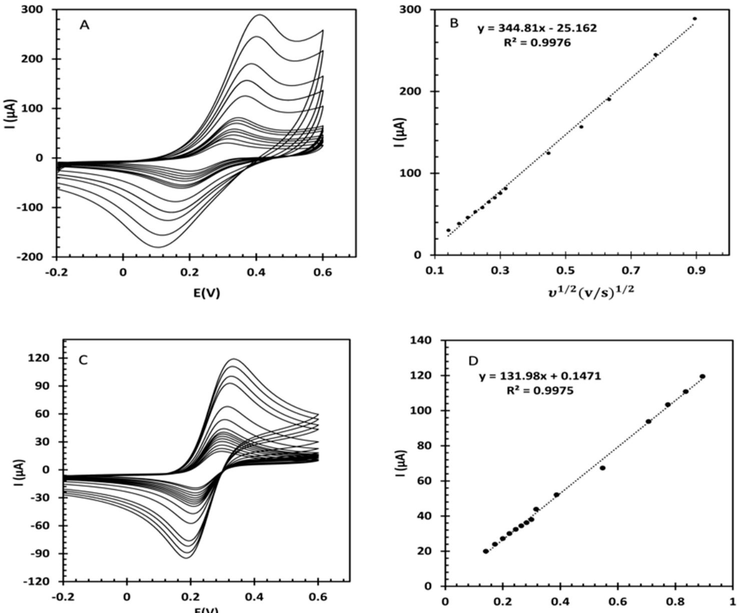

Figure S1. CVs of fl-NiO/C/GCE in 5.0 mM $[Fe(CN)_6]^{3-/4-}$ in 0.1 M KCl at a scan rate of 20–800 mv/s (**A**) and the plot of I versus $\upsilon^{1/2}$ (**B**). CVs of bare GCE in 5.0 mM $[Fe(CN)_6]^{3-/4-}$ in 0.1 M KCl at a scan rate of 20–800 mv/s (**C**) and the plot of I versus $\upsilon^{1/2}$ (**D**).

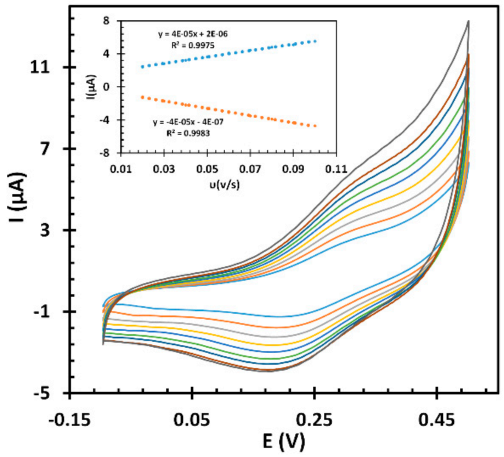

Figure S2. Cyclic voltammogram of fl-NiO/C/GCE in 1M NaOH solution at various scan rate 10–100mV/s. (Inset: Plot of I versus υ)

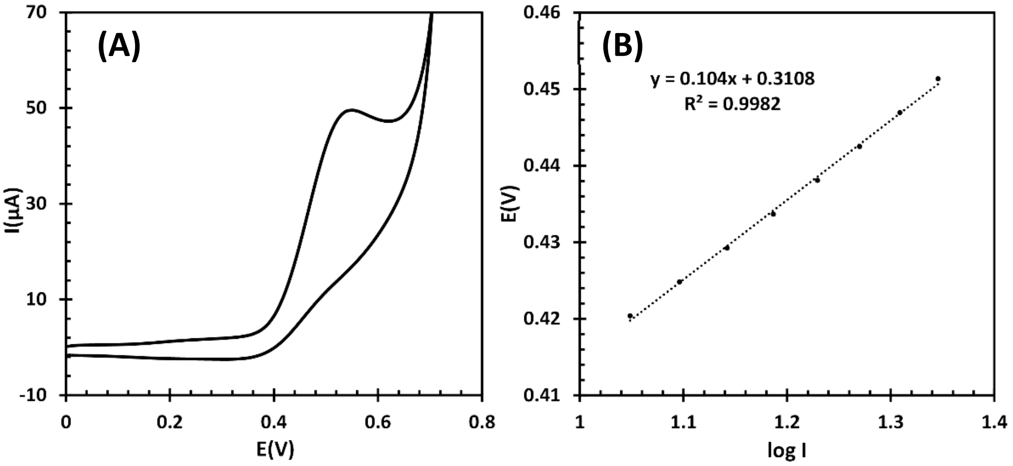

Figure S3. CVs of fl-NiO/C/GCE in 1mM LGA in 1 M NaOH at a scan rate of 10 mV/s (**A**). Tafel plot E vs log I (**B**).

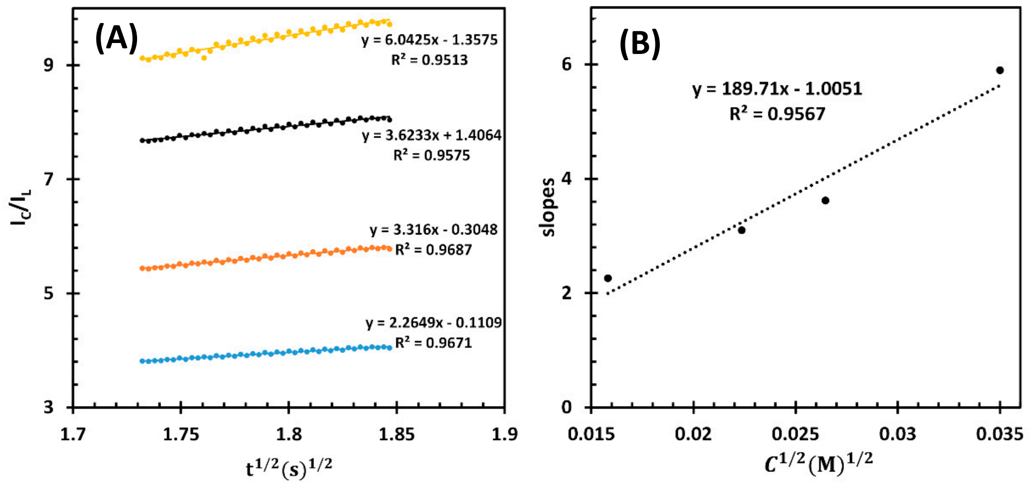

Figure S4. The Ic/IL vs. $t^{1/2}$ plot at different concentrations of LGA (250, 500, 700, and 1000 μM) (**A**). The plot of the slope of the (Ic/IL vs.$t^{1/2}$) vs. $C^{1/2}$ (**B**).

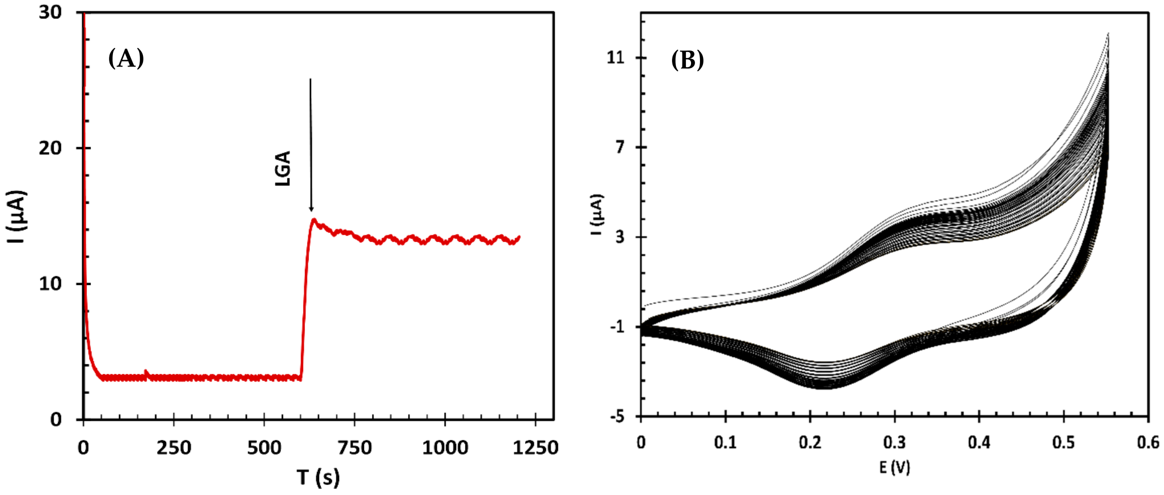

Figure S5. Amprometric response of fl-NiO/C/GCE 600 second before and after injection of 200μL GLA (**A**). CVs of fl-NiO/C/GCE in 1 M NaOH without LGA for 100 cycles, scan rate 50 mv/s (**B**).

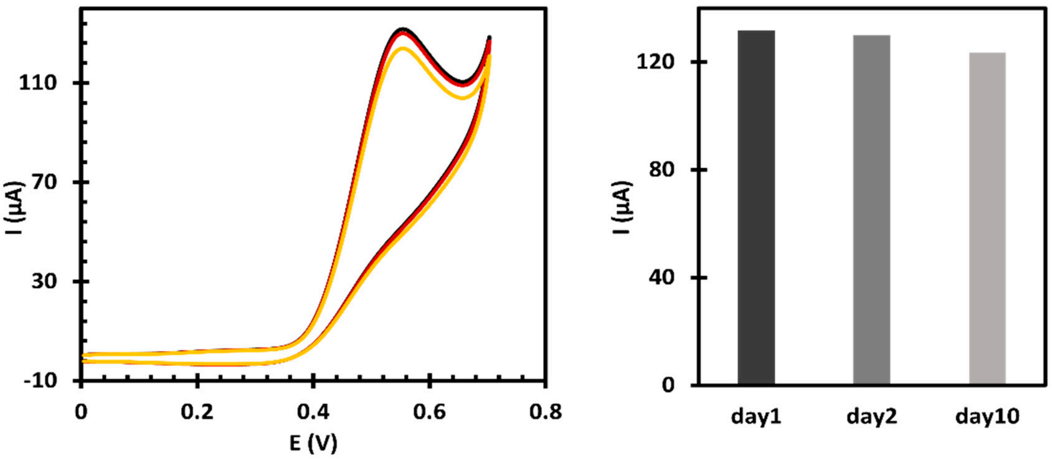

Figure S6. Stability of the fl-NiO/C/GCE during different times.

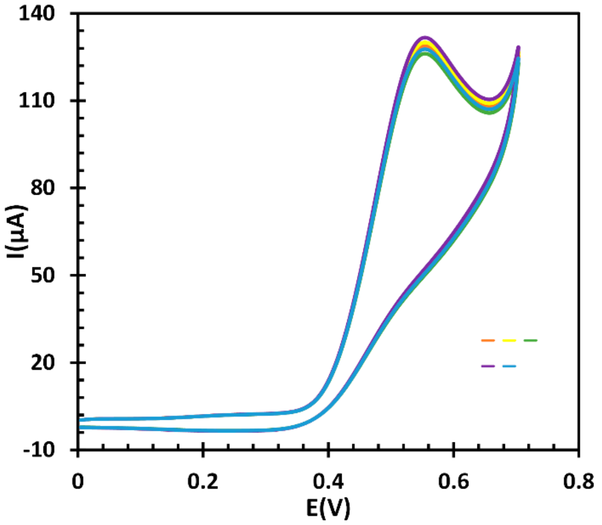

Figure S7. CVs of 5 prepared fl-NiO/C/GCE in 1mM LGA in 1 M NaOH at a scan rate of 50 mV/s.

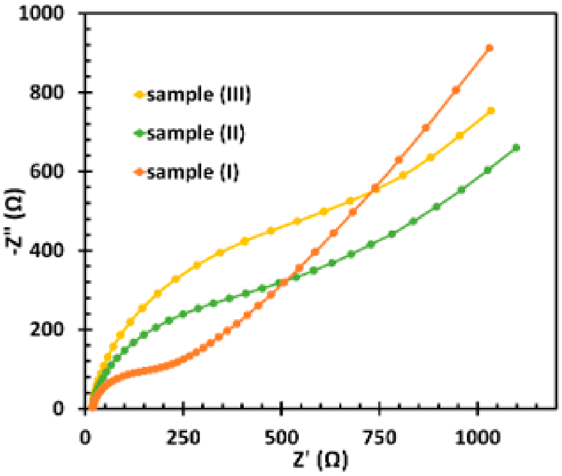

Figure S8. EIS responses of the fl-NiO/C/GCE towards sample (I–III).
